# 16p11.2 deletion mice exhibit compromised fronto-temporal connectivity, GABAergic dysfunction, and enhanced attentional ability

**DOI:** 10.1038/s42003-023-04891-2

**Published:** 2023-05-24

**Authors:** Rebecca L. Openshaw, David M. Thomson, Greg C. Bristow, Emma J. Mitchell, Judith A. Pratt, Brian J. Morris, Neil Dawson

**Affiliations:** 1 School of Psychology and Neuroscience, College of Medical, Veterinary and Life Sciences, University of Glasgow, Sir James Black Building, Glasgow, G12 8QQ UK; 2 Strathclyde Institute of Pharmacy and Biomedical Sciences, University of Strathclyde, Glasgow, G4 0RE UK; 3 Department of Biomedical and Life Sciences, Lancaster University, Lancaster, LA1 4YW UK; 4 Present Address: School of Pharmacy and Medical Sciences, University of Bradford, Bradford, BD7 1DP UK

**Keywords:** Autism spectrum disorders, Attention

## Abstract

Autism spectrum disorders are more common in males, and have a substantial genetic component. Chromosomal 16p11.2 deletions in particular carry strong genetic risk for autism, yet their neurobiological impact is poorly characterised, particularly at the integrated systems level. Here we show that mice reproducing this deletion (16p11.2 DEL mice) have reduced GABAergic interneuron gene expression (decreased parvalbumin mRNA in orbitofrontal cortex, and male-specific decreases in Gad67 mRNA in parietal and insular cortex and medial septum). Metabolic activity was increased in medial septum, and in its efferent targets: mammillary body and (males only) subiculum. Functional connectivity was altered between orbitofrontal, insular and auditory cortex, and between septum and hippocampus/subiculum. Consistent with this circuit dysfunction, 16p11.2 DEL mice showed reduced prepulse inhibition, but enhanced performance in the continuous performance test of attentional ability. Level 1 autistic individuals show similarly heightened performance in the equivalent human test, also associated with parietal, insular-orbitofrontal and septo-subicular dysfunction. The data implicate cortical and septal GABAergic dysfunction, and resulting connectivity changes, as the cause of pre-attentional and attentional changes in autism.

## Abstract (web summary)

Mice hemizygous for the 16p11.2 deletion exhibit localized functional dysconnectivity and altered GABAergic gene expression, implicating GABAergic dysfunction and resulting connectivity changes in attentional changes in autism spectrum disorder.

## Introduction

Autism spectrum disorders (ASD) are a heterogeneous group of related neurodevelopmental disorders characterised by social dysfunction, communication difficulties, repetitive behaviour, restricted interests and altered sensory perception. Interestingly, cognitive abilities range from profound intellectual disability (ID) to enhanced performance in some cognitive domains^1,2^. In recent years, an increase in the prevalence of ASD has been observed, with current estimates of ~1 in 100 people affected. Boys are around 4 times more likely to be affected than girls, although this gap is narrowing^3–5^. There are currently no effective treatments for ASD. There is a strong genetic component to the chance of developing ASD, with heritability estimates of up to 90%^6^. Typically, multiple common sequence variations of small effects interact to increase the chance of developing ASD, in combination with environmental risk factors. However, in addition to these common genetic variants, rare variants also exist that individually have a much greater effect. These rare variants can be genetically reproduced in mice, providing valuable insight into the neurobiological dysfunction that underlies ASD. If translationally-relevant phenotypes and endophenotypes can be identified, these mouse models will ultimately provide a means to identify and test novel treatment strategies for aspects of ASD.

Carriers of a ~29 gene deletion on chr.16p11.2 are at greatly increased chance (~35 times) of developing ASD^7–11^ and related conditions with shared heritability, such as attention deficit hyperactivity disorder (ADHD) and anxiety disorders^12^. 16p11.2 deletions are one of the most prevalent causes of syndromic ASD and hence are of great interest for further understanding the neurobiological dysfunction that underlies ASD symptomatology.

Given the heterogeneity of ASD, it is not surprising that structural and functional neuroimaging studies have identified many affected brain regions and networks. These include changes in fronto-temporal and frontoparietal regions, the amygdala-hippocampal complex, cerebellum, basal ganglia and cingulate cortex^13–16^. These regions form part of functional brain networks including the Default Mode Network (DMN), the Salience Network (SalN) and the Visual Attention Network (VAN). These networks, and the interactions between them, are dysfunctional in ASD. ASD is associated with both hypoconnectivity and hyperconnectivity of these macroscale brain networks, whereas connectivity in local circuits is increased^16–19^. According to current theories, this altered connectivity sits alongside, and is possibly caused by, GABAergic interneuron and glutamate system dysfunction. Excitation/inhibition (E/I) imbalance in ASD, with compromised function of local inhibitory GABAergic interneurons in cortical areas, is thought to disrupt the balance of intrinsic and extrinsic regional network activity^20^. While certain symptoms of ASD have been broadly correlated with particular networks, hallmark connectivity patterns remain unclear.

Adoption of multi-scale and integrative (endo)phenotyping, along with effective forward and reverse translational approaches, is key to understanding ASD and identifying translational biomarkers for diagnosis, prognosis and treatment validation^21^. The identification of 16p11.2 deletions as a major risk factor for ASD provides an opportunity to reverse translate into preclinical studies, in order to gain an increased understanding of the neurobiological mechanisms involved in ASD. In addition, translational data-driven preclinical brain imaging approaches can be used to identify cross-species commonalities in brain dysfunction, as well as reveal hitherto unknown brain network impairments in ASD^22^.

Here we undertake a multi-scale and integrative characterisation of CNS function in mice reproducing the 16p11.2 deletion, with a focus on characterising translationally-relevant perturbations. Thus we characterise ASD-relevant alterations in GABAergic interneuron gene expression and related functional brain imaging changes, in terms of regional metabolism and data-driven functional brain network connectivity changes. Guided by our observations in these endophenotypic measures we prioritised the characterisation of behaviours dependent on the brain regions and networks we identified as being dysfunctional in 16p11.2 DEL mice. Thus, we characterise the performance of 16p11.2 DEL mice in behavioural translational measures known to be impacted in ASD – pre-attentional sensorimotor gating, measured using prepulse inhibition (PPI) of the startle reflex, and attentional performance, in the visual rodent continuous performance task (rCPT) utilising touchscreen apparatus designed to reproduce the widely employed human CPT^23^. There are conflicting reports on whether attentional processing is enhanced or impaired in ASD individuals. However, there is emerging evidence to support an enhanced capacity for selective attention in some contexts^1,24–26^.

## Results

### Functional brain imaging identifies widespread dysfunctional integration between diverse neural systems in 16p11.2 DEL mice

**Fig. 1:**
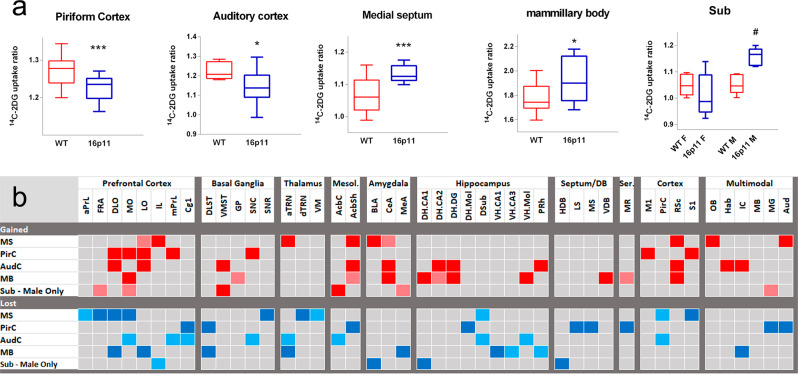
Overt alterations in brain region metabolism and altered inter-regional functional connectivity in 16p11.2 DEL mice. **a** 16p11.2 DEL mice have decreased activity in the piriform (PirC, *p* = 0.008) and auditory (AudC, *p* = 0.014) cortex with increased metabolic activity in the medial septum (MS, *p* = 0.001) and mammillary body (MB, *p* = 0.014). The impact of 16p11.2 deletion on metabolism in these regions was not significantly influenced by sex, so the data are pooled for both sexes. Increased functional activity in the hippocampal subiculum (Sub) was found only in males (sex x genotype interaction: *p* = 0.03, ANOVA). *P < 0.05 and ***P < 0.001 difference from WT (ANOVA, main effect of genotype). ^#^*P* < 0.05 difference from same sex WT (Tukey’s HSD). Box plots show median and interquartile range with “Tukey” whiskers. **b** Heatmap showing the altered inter-regional connectivity of regions with altered metabolism in 16p11.2 DEL mice, determined using PLSR. Red denotes inter-regional connectivity present in 16p11.2 DEL but not WT mice (VIP 95% CI > 1.0 in 16p11.2 DEL and <1.0 in WT). Light red denotes *z* > 1.96 and dark red *z* > 2.58 difference between genotypes. Blue denotes lost inter-regional connectivity in 16p11.2 DEL mice (VIP 95% CI < 1.0 in 16p11.2 DEL and >1.0 in WT). Light blue denotes *z* > 1.96 and dark blue denotes *z* > 2.58 difference between genotyes. Data for Sub show connectivity changes selectively found in 16p11.2 DEL male mice that are not found in 16p11.2 DEL female mice. *n* = 12 (6 male) mice/genotype. *Prefrontal cortex*: aPrL Anterior Prelimbic Cortex, FRA Frontal Association Area, DLO Dorsolateral Orbital Cortex, MO Medial Orbital Cortex, LO Lateral Orbital Cortex, mPrL Medial Prelimbic Cortex, IL Infralimbic Cortex, Cg1 Cingulate Cortex; Cortex: Ins Insular Cortex, Piri Piriform Cortex, RSC Retrosplenial Cortex, S1 Somatosensory Cortex; *Thalamus*: AM Anteromedial Thalamus, TRN Thalamic Reticular Nucleus, aRT Anterior Reticular Thalamus, AV Anteroventral Thalamus, MD Mediodorsal Thalamus, VL Ventrolateral Thalamus, VM Ventromedial Thalamus; *Mesolimbic*: AcbC Nucleus Accumbens Core, AcbSh Nucleus Accumbens Shell, VTA ventral Tegmental Area; Basal Ganglia: DLST Dorsolateral Striatum, VMST Ventromedial Striatum, GP Globus Pallidus, SNC Substantia Nigra pars Compacta, SNR Substantia Nigra pars Reticulata; *Amygdala*: BLA Basolateral Amygdala, CeA Central Amygdala, MeA Medial Amygdala; *Auditory*: IC Inferior Colliculus, MG Medial Geniculate, DB Septum/Diagonal Band of Broca, LS Lateral Septum, MS Medial Septum, HDB Horizontal Limb of DB, VDB Vertical Limb of DB; *Hippocampus*: DSub Dorsal Subiculum, DH CA1 Dorsal Cornu Ammonis 1, DH CA2 Dorsal Cornu Ammonis 2, DH Mol Dorsal Molecular Layer, DH DG Dorsal Dentate Gyrus, VH CA1 Ventral Cornu Ammonis 1, VH CA3 Ventral Cornu Ammonis 3, VH Mol Ventral Molecular Layer, VH DG Ventral Dentate Gyrus; *Multimodal*: Hab Habenula, MB Mammillary Body. Regional abbreviations are also shown in the supplemental material (Table S3).

Most CNS regions showed unchanged metabolic activity in 16p11.2 DEL mice, as evidenced by unaltered ^14^C-2-DG metabolism. However, metabolism in the piriform (PirC) and auditory cortex (AudC) was reduced in 16p11.2 DEL mice. In contrast, metabolic activity was increased in the medial septum (MS) and mammillary body (MB) (Fig. 1a). These effects were not influenced by sex. However, we also found markedly elevated metabolism in the hippocampal subiculum (Sub) of 16p11.2 DEL mice, but only in males (Fig. 1a).

To elucidate the dysfunctional inter-regional connectivity of these regions in 16p11.2 DEL mice we employed Partial Least Squares Regression (PLSR) analysis (Fig. 1b). We found evidence for both gained and lost inter-regional functional connectivity for each region, supporting widespread neural systems dysconnectivity in 16p11.2 DEL mice. For the MS, functional connectivity was gained to amygdala (BLA, CeA), retrosplenial (RSC) and AudC. Altered MS functional connectivity to the prefrontal cortex (PFC) was complex, with gained connectivity to the infralimbic (IL) and lateral orbital (LO) cortical subfields, but lost connectivity to other PFC subfields (anterior prelimbic (aPrL), frontal association cortex (FRA), DLO, MO) and to the hippocampal Sub. We also found evidence for both gained and lost connectivity with the PFC, on a subfield-dependent basis, when the AudC and PirC were considered as seed regions in the analysis. This included gained functional connectivity to orbital cortex subfields (DLO, MO, LO) but lost connectivity to cingulate cortex (Cg1) in 16p11.2 DEL mice. Interestingly, there was also evidence for dysfunctional connectivity between the AudC and hippocampus in 16p11.2 DEL mice, with gained connectivity to the dorsal hippocampus (DHCA2, DH.DG) but lost connectivity to the Sub of the ventral hippocampus. A similar pattern of hippocampal dysconnectivity was also observed for the MB, with lost connectivity to ventral hippocampus (VH.CA1, VH.CA3) but enhanced connectivity to dorsal hippocampus (DH.CA1, DH.CA2, DH.DG) in 16p11.2 DEL mice.

Given that hippocampal Sub activity was selectively increased in males, we characterised the alterations in Sub connectivity seen selectively in male 16p11.2 DEL animals. Again we found evidence for complex dysfunctional connectivity to the PFC, with both gained (FRA, MO) and lost (IL) connectivity supported on a PFC subfield-dependent basis.

Overall these data support complex dysfunctional connectivity between a diverse range of neural systems in 16p11.2 DEL mice, with notably altered prefrontal (PrL, IL, LO, DLO, MO), hippocampal and temporal cortex (PirC, AudC) functional connectivity. In addition, dysfunctional connectivity with closely-related subcortical areas including the amygdala, thalamus, septum and mammillary body was also found.

### Brain network hub dysconnectivity in 16p11.2 DEL mice supports altered PFC, septum and insular cortex connectivity

Given the complex inter-regional dysconnectivity seen in 16p11.2 DEL mice (Fig. 1) we next sought to characterise the alterations in brain network hub functional connectivity in these animals, using centrality analysis. This identified the PFC, FRA and the insular cortex (Ins) as important functional hubs in the brain networks of WT mice, that lost their hub status in 16p11.2 DEL mice. In addition, the dentate gyrus of the dorsal hippocampus (DH.DG) lost its hub status, as did the vertical limb of the diagonal band of broca (VDB), part of the septum/DB system and the ventromedial thalamic (VM) nucleus (Table 1). Only one region, the horizontal limb of the diagonal band of Broca (HDB), also part of the septum/DB, was identified as a region that gained hub status, indicating the increased connectivity of this region in the brain networks of 16p11.2 DEL mice. The loss of network hubs in frontal and temporal cortex, and the reciprocal changes in septum/DB, suggest a reorganisation of fronto-temporal and associated subcortical circuit connectivity in 16p11.2 DEL mice.

**Table 1:** Altered hub regions in the functional brain networks of 16p11.2 DEL mice.

Region	Wild-type	16p11.2 DEL	Measure
Frontal association cortex (FRA)	**6.00**	−1.05*	B_c_
Insular cortex (Ins)	**7.96**	1.10*	B_c_
Verticle limb diagonal band of broca (VDB)	**2.57**	−2.34*	K_i_
Horizontal limb diagonal band of broca (HDB)	−1.61	5.01*	B_c_
Ventromedial thalamus (VM)	**5.27**	−0.98*	B_c_
Dorsal hippocampus-dentate gyrus (DH.DG)	**4.68**	−0.88*	B_c_

Data shown as the standardised *z* score relative to 11,000 random calibrated Erdös-Rényi networks. Z > 1.96 (bold) denotes a significant hub in the real brain network. *denotes *P* < 0.05 significant difference from WT (55,000 data permutations). *Bc* betweeness centrality, *Ki* degree centrality.

**Fig. 2:**
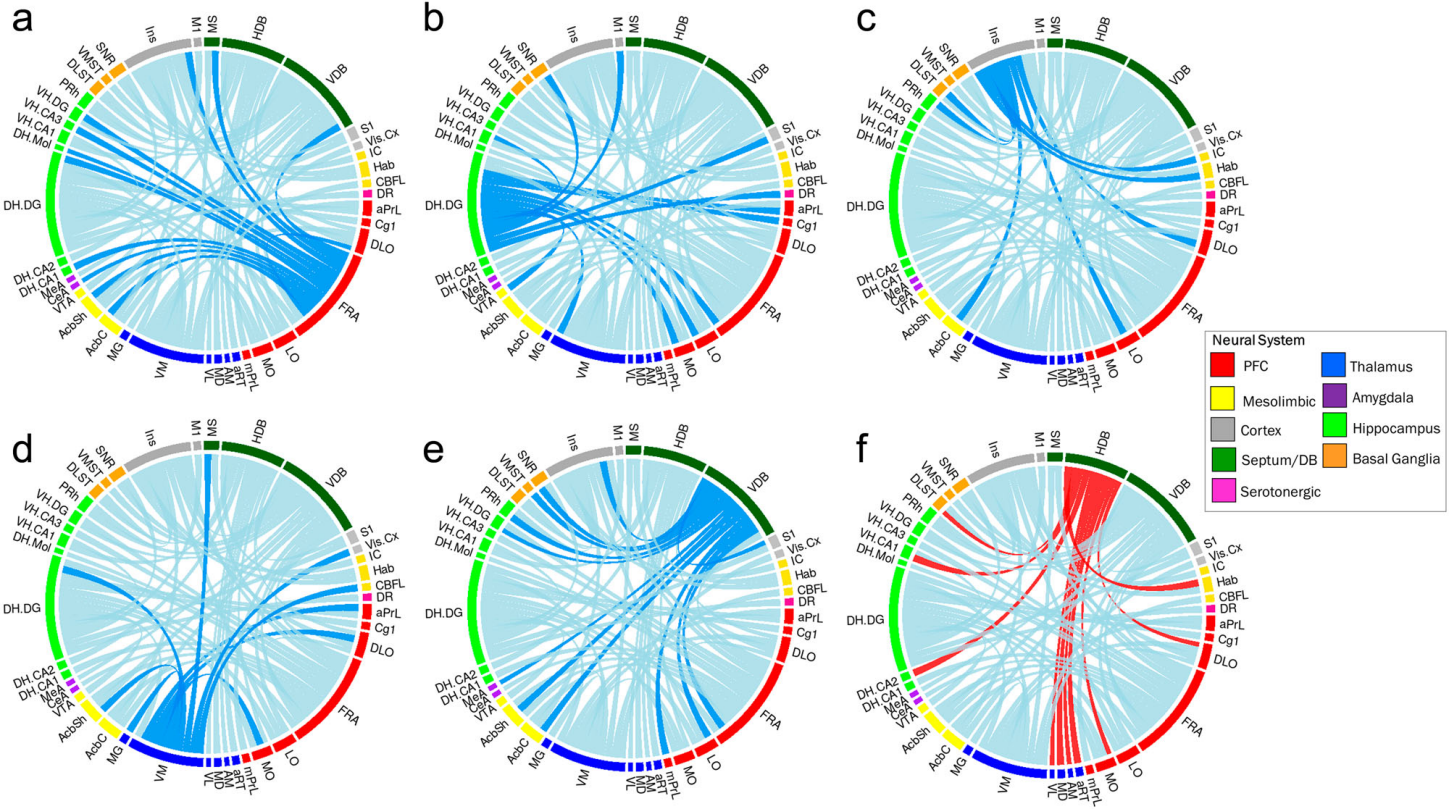
Altered inter-regional connectivity underlies the altered hub region connectivity seen in the functional brain networks of 16p11.2 DEL mice. Chord diagrams showing lost (dark blue) and gained (red) inter-regional connectivity for the **a** Frontal Association Cortex (FRA), **b** Dorsal Hippocampus Dentate Gyrus (DH DG) **c** Insular Cortex (Ins) **d** Ventromedial Thalamus (VM) in **e** Vertical Limb of the Diagonal Band of Broca (VDB) and **f** Horizontal Limb of the Diagonal Band of Broca (HDB) in 16p11.2 DEL mice. Dark blue denotes significantly lost inter-regional connectivity (VIP 95% CI < 1.0 in 16p11.2 DEL and >1.0 in WT; *z* <−1.96 in DELs v WT) and red denotes significantly gained connectivity (VIP 95% CI > 1.0 16p11.2 DEL and <1.0 WT; z > 1.96 in DELs v WT) in 16p11.2 DEL mice. *n* = 12 (6 male) mice/genotype. *Prefrontal cortex*: aPrL Anterior Prelimbic Cortex, FRA Frontal Association Area, DLO Dorsolateral Orbital Cortex, MO Medial Orbital Cortex, LO Lateral Orbital Cortex, mPrL Medial Prelimbic Cortex, IL Infralimbic Cortex, Cg1 Cingulate Cortex; Cortex: Ins Insular Cortex, Piri Piriform Cortex, RSC Retrosplenial Cortex, S1 Somatosensory Cortex; *Thalamus*: AM Anteromedial Thalamus, TRN Thalamic Reticular Nucleus, aRT Anterior Reticular Thalamus, AV Anteroventral Thalamus, MD Mediodorsal Thalamus, VL Ventrolateral Thalamus, VM Ventromedial Thalamus; Mesolimbic: AcbC Nucleus Accumbens Core, AcbSh Nucleus Accumbens Shell, VTA ventral Tegmental Area; Basal Ganglia: DLST Dorsolateral Striatum, VMST Ventromedial Striatum, GP Globus Pallidus, SNC Substantia Nigra pars Compacta, SNR Substantia Nigra pars Reticulata; *Amygdala*: BLA Basolateral Amygdala, CeA Central Amygdala, MeA Medial Amygdala; *Auditory*: IC Inferior Colliculus, MG Medial Geniculate, DB Septum/Diagonal Band of Broca, LS Lateral Septum, MS Medial Septum, HDB Horizontal Limb of DB, VDB Vertical Limb of DB; *Hippocampus*: DSub Dorsal Subiculum, DH CA1 Dorsal Cornu Ammonis 1, DH CA2 Dorsal Cornu Ammonis 2, DH Mol Dorsal Molecular Layer, DH DG Dorsal Dentate Gyrus, VH CA1 Ventral Cornu Ammonis 1, VH CA3 Ventral Cornu Ammonis 3, VH Mol Ventral Molecular Layer, VH DG Ventral Dentate Gyrus; *Multimodal*: Hab Habenula, MB Mammillary Body. Brain region abbreviations are also shown in the supplemental material (Supplementary Table 3). Neural systems are colour coded and indicated in the Figure legend.

To characterise the altered inter-regional connectivity that underlies the altered hub status of these regions, we employed PLSR analysis, using the regions showing altered centrality as the seed brain regions in this analysis^27–29^. Again, these results supported dysfunctional PFC and orbitofrontal cortex (OFC) connectivity in 16p11.2 DEL mice. Lost PFC/OFC-hippocampal (FRA and DH.DG seeds), PFC-septum (FRA and VDB seeds), PFC/OFC-thalamic (VM seed) and PFC-accumbens (FRA seed) functional connectivity was evident in 16p11.2 DEL mice (Fig. 2a–f).

Insular and septum/DB dysconnectivity was further supported in 16p11.2 mice in this analysis. Inter-regional dysconnectivity between the Ins and the OFC, perirhinal cortex (PRh) and accumbens/striatum was found (Fig. 2c). When the VDB was considered as the seed region, compromised septum-hippocampal and septum-accumbens connectivity was identified (Fig. 2e). By contrast, when the HDB was considered as the seed region, gained septum-thalamic, septum-OFC and septum-hippocampal connectivity was detected in 16p11.2 DEL mice (Fig. 2f). Interestingly, there was no overlap between the PFC and hippocampal subfields showing lost connectivity to the septum VDB and those showing gained connectivity to the septum HDB. Overall, these data support a complex, region-specific reorganisation of septum connectivity to the PFC and hippocampus in 16p11.2 DEL mice.

### GABAergic interneuron gene expression is altered in brain regions impacted by 16p11.2 deletion

**Fig. 3:**
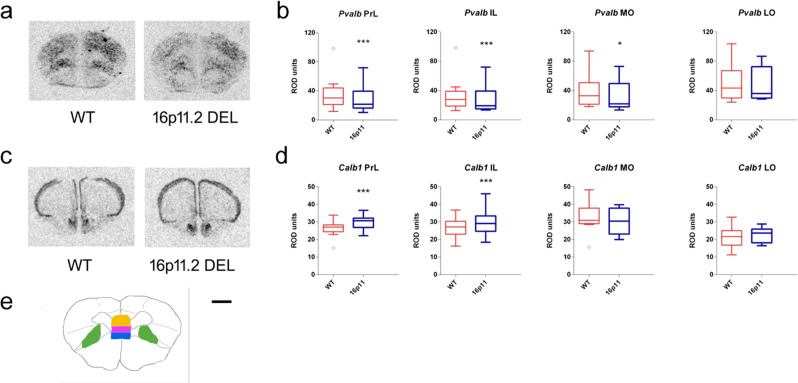
16p11.2 DEL mice have decreased parvalbumin (*Pvalb*) and increased calbindin (*Calb1*) mRNA expression in the prefrontal (PFC) and orbital (OFC) cortex. Representative autoradiographs for **a** *Pvalb* and **c** *Calb1* mRNA expression. **b** *Pvalb* expression is decreased in the prelimbic cortex (PrL), infralimbic cortex (IL) and medial orbital (MO) cortex, but not the lateral orbital (LO) cortex of 16p11.2 DEL mice. **d** *Calb1* expression is increased in the medial prefrontal (PrL and IL), but not the orbital (MO and LO) regions in 16p11.2 DEL mice. **e** location of regions analysed: PrL (orange), IL (purple), MO (blue) and LO (green). Scale bar represents 750 μm. Box plots show median and interquartile range with “Tukey” whiskers. WT = wild-type mice; 16p11.2 DEL = 16p11.2 deletion mice. Data are pooled for sex, as no significant sex x genotype interactions were found. Data were analysed by ANOVA; *F* and *p* values are provided in Supplementary Table 1. *P < 0.05, ***P < 0.001, ANOVA main effect of genotype. *n* = 12 (8 male) mice/genotype.

Since ASD is associated with GABAergic interneuron dysfunction and alterations in GABAergic interneuron gene expression^30,31^, we hypothesised that the perturbations in regional and network connectivity detected in 16p11.2 DEL mice may, in part, reflect localised compromised inhibitory interneuron function. Indeed, ASD is associated with reductions in GABAergic interneuron markers in cortical areas shown to be dysfunctional in 16p11.2 DEL mice, such as PFC/OFC and parietal cortex (ParC)^32^. We, therefore, examined the expression of mRNAs for *Gad1*, encoding the GABA synthetic enzyme Gad67, and the interneuron subtype-specific activity-dependent markers parvalbumin (*Pvalb*), calbindin (*Calb1*), calretinin (*Calb2*) and somatostatin (*Sst*), in 16p11.2 DEL mice. We observed reduced *Pvalb* expression in multiple PFC/OFC subfields including the prelimbic (PrL), infralimbic (IL) and medial orbital (MO) cortex (Fig. 3a, b, e). In contrast, *Calb1* expression was elevated in the PFC (PrL and IL subfields) (Fig. 3c–e). We found no evidence that the impact of 16p11.2 DEL on GABAergic gene expression was significantly influenced by sex in these PFC/OFC regions.

The connectivity analysis identified altered connectivity between the OFC and septum/DB system and between the OFC and Ins in 16p11.2 DEL mice. We therefore also examined GABAergic gene expression in the septum and Ins.

**Fig. 4:**
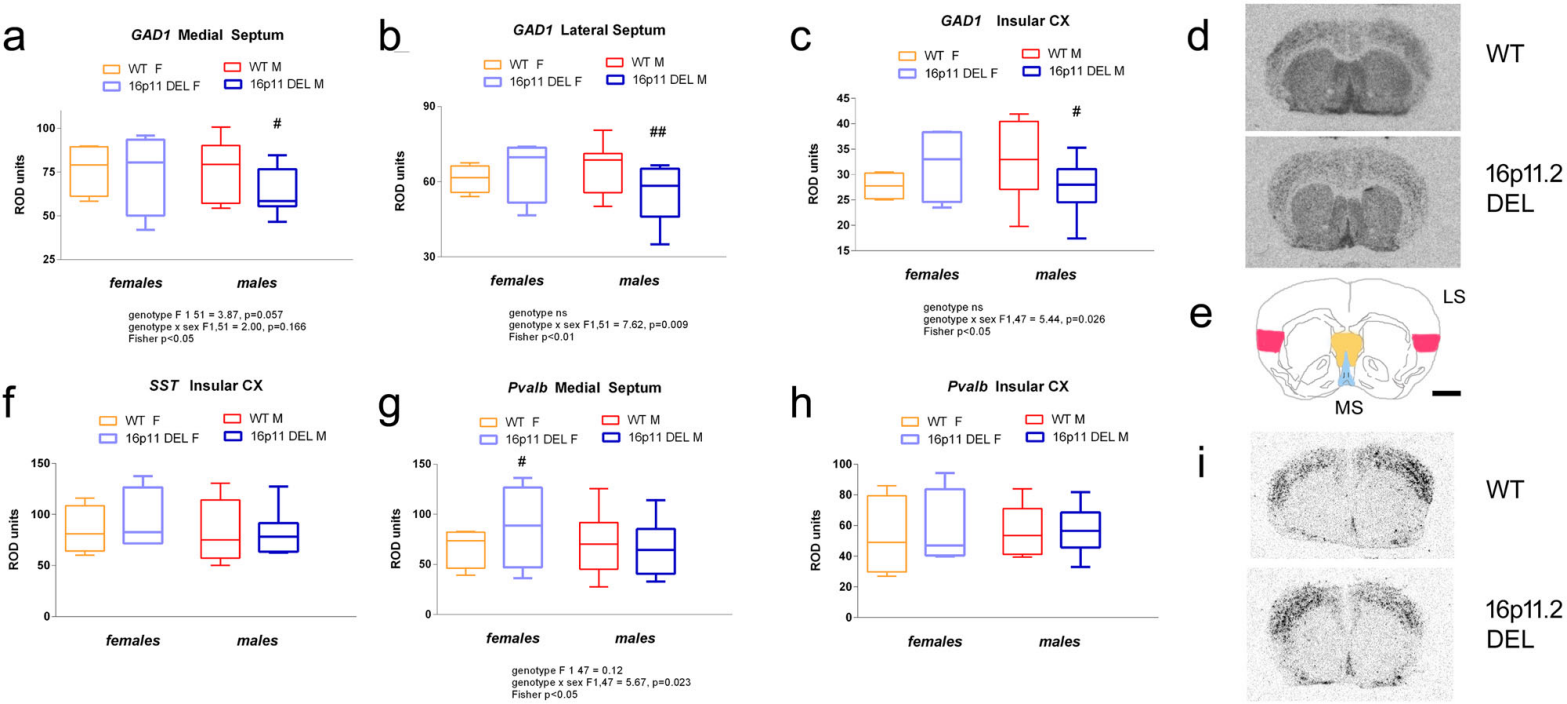
Male, but not female, 16p11.2 DEL mice have decreased GABAergic gene expression in the septum and insular cortex. *Gad1* (*Gad67*) mRNA expression is decreased in the **a** medial septum, **b** lateral septum and **c** insular cortex in male 16p11.2 DEL mice. *Sst* mRNA expression is not altered in the **f** insular cortex of 16p11.2 DEL mice. *Pvalb* expression **g** is increased in female 16p11.2 DEL mice in the medial septum, but **h** unaltered in the insular cortex of 16p11.2 DEL mice. Representative autoradiographs for **d** *Gad1* and **i** *Pvalb* expression in male mice. **e** Location of regions analysed: LS (orange), MS (blue) and Insula (red). Scale bar represents 1000 μm. Box plots show median and interquartile range with “Tukey” whiskers. WT F = wild-type female mice; WT M = wild-type male mice; 16p11 DEL F = 16p11.2 deletion female mice; 16p11.2 DEL M = 16p11.2 deletion male mice. Data were analysed by ANOVA. *F* and *p* values are provided in Supplementary Table 1. ^#^*P* < 0.05, ^##^*P* < 0.01, Fisher’s post hoc test, *n* = 12 (8 male) mice/genotype.

**Fig. 5:**
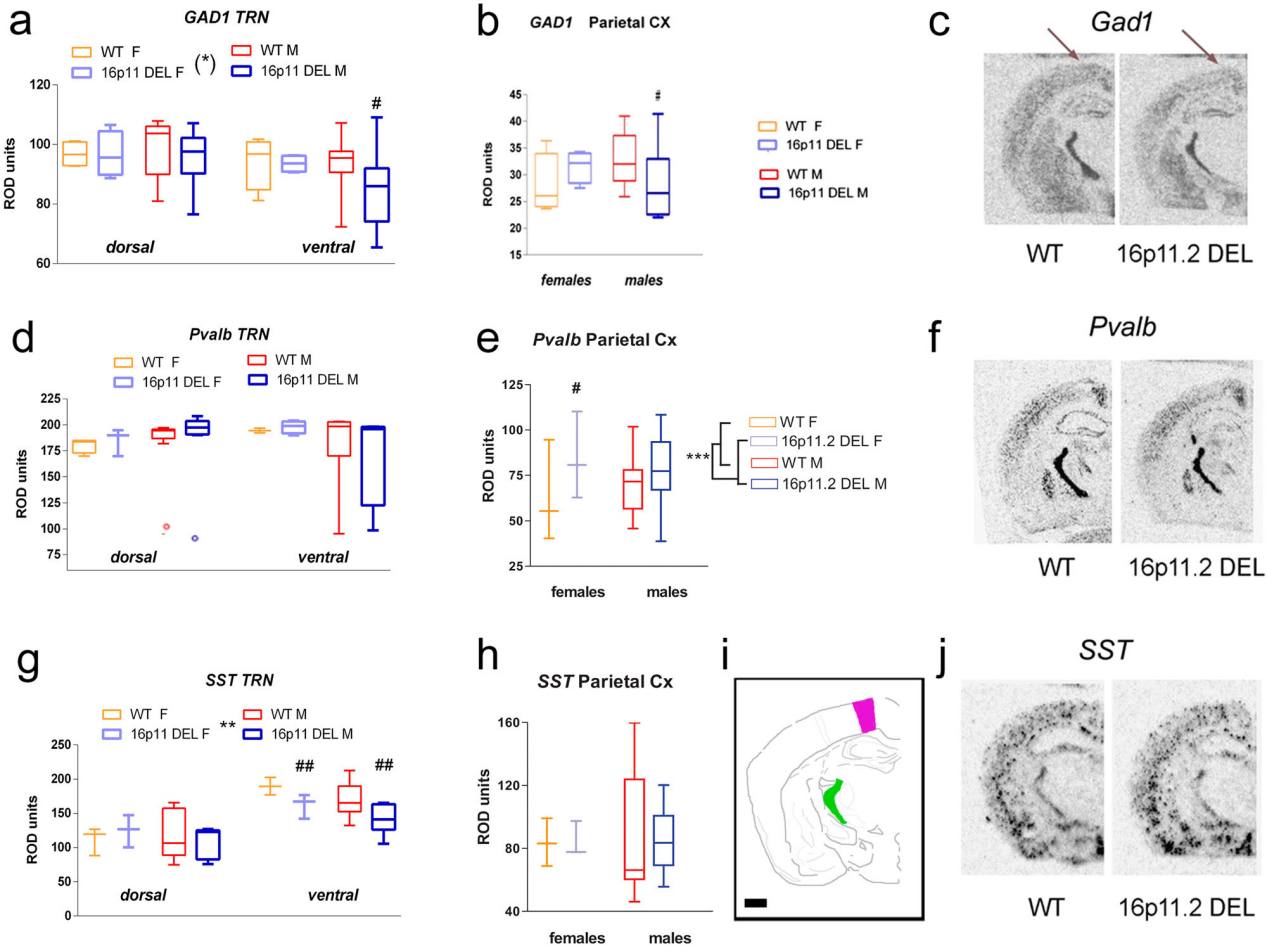
Male, but not female, 16p11.2 DEL mice show reduced GABAergic gene expression in the reticular thalamic nucleus (TRN) and parietal cortex (ParC). Levels of *Gad1* mRNA in the **a** TRN and **b** ParC are reduced in 16p11.2 DEL male mice. *Pvalb* mRNA expression is unaltered in the **d** TRN, but increased in the **e** ParC of 16p11.2 mice of both sexes. *Sst* mRNA expression is decreased in the **g** TRN and unaltered in the **h** ParC of 16p11.2 DEL male mice. Representative autoradiographs for **c** *Gad1*, **f** *Pvalb* and **j** *Sst* mRNA expression in male mice. **i** Location of regions analysed: TRN (green) and ParC (purple). Scale bar represents 1000 μm. Box plots show median and interquartile range with “Tukey” whiskers. WT F = wild-type female mice; WT M = wild-type male mice; 16p11 DEL F = 16p11.2 deletion female mice; 16p11.2 DEL M = 16p11.2 deletion male mice. Data were analysed by ANOVA. F and *p* values are provided in Supplementary Table 1. ***P < 0.001 main effect of genotype (ANOVA). ^#^*P* < 0.05, ^##^*P* < 0.01, Tukey’s *post-hoc* test vs same sex WT group. *n* = 12 (8 male) mice/genotype. Arrows in **c** also show ParC location of *Gad1* expression.

In the septum, clear sex-dependent alterations in GABAergic gene expression were detected. Male, but not female, 16p11.2 DEL mice showed reduced *Gad1* expression in MS and LS (Fig. 4a, b, d, e). By contrast, female, but not male, 16p11.2 DEL mice showed elevated *Pvalb* expression in the MS (Fig. 4e, g, i). Conversely, Male-specific *Gad1* reductions were also observed in the Ins (Fig. 4c), while *Sst* and *Pvalb* expression were unaltered in 16p11.2 DEL mice of both sexes (Fig. 4f, h). Similarly *Gad1* expression levels were also selectively decreased in the ParC of male 16p11.2 DEL mice (Fig. 5b, c, i). Conversely, *Pvalb* expression in ParC was increased in 16p11.2 DEL mice, independent of sex (Fig. 5e, f) while *Sst* expression was unaltered (Fig. 5h, j). Somewhat surprisingly, no differences in GABAergic gene expression were detected in the AudC or PirC, where metabolic activity was previously identified as being reduced in 16p11.2 DEL mice (Fig. 1, Supplementary Table 1)

The dysfunction of thalamocortical loops is a feature of a number of neurodevelopmental disorders, including ASD^33^. The thalamic reticular nucleus (TRN) is a key regulator of all thalamocortical loops^34^, and of the mediodorsal thalamus—PFC loop in particular^35^. Given the TRN dysconnectivity found in 16p11.2 DEL mice (Figs. 1 and 2), the GABAergic dysfunction characterised in other brain regions in these animals (Figs. 3–5) and the high density of GABA interneurons in this structure, we also investigated GABAergic gene expression in the TRN.

Again, there was a male-specific reduction in *Gad1* expression in the TRN (Fig. 5a, c, i), while *Sst* expression was reduced in 16p11.2 DEL mice of both sexes (Fig. 5g, i, j) and *Pvalb* expression levels were unaltered (Fig. 5d, f).

### 16p11.2 DEL mice show impaired pre-attentional filtering and enhanced attentional processing

Our brain connectivity and GABAergic gene expression studies in 16p11.2 DEL mice highlighted a complex and widespread dysregulation of neuronal circuitry implicated in behavioural changes relevant to ASD, such as repetitive behaviours^36^, social difficulties^37,38^, pre-attentional filtering (PPI) and attentional processing. This includes dysfunction of the PFC/OFC, septum and TRN. PPI involves a diffuse neural circuit with a core component of AudC, PrL, ParC, Ins, TRN, septum, hippocampus, habenula and inferior colliculi^39,40^, while increased attentional demands recruit circuitry centred on Ins, PrL, OFC, cingulate, ParC, MS, Sub and mammillary bodies^41–43^. Therefore, to elucidate the behavioural impact of these molecular and systems-level alterations, and to assess alignment with ASD-relevant deficits, we tested 16p11.2 DEL mice in the PPI and rCPT translational paradigms.

**Fig. 6:**
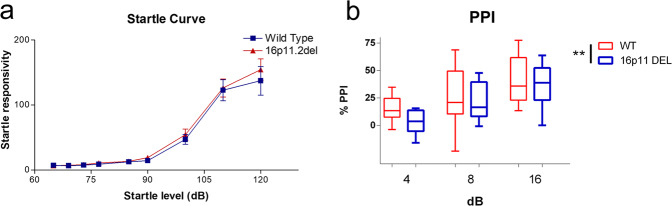
16p11.2 DEL mice show decreased PPI. **a** The startle reflex is unaltered but **b** PPI is significantly reduced in 16p11.2 DEL mice. **a** Data shown as mean ± s.e.m. **b** Box plots show median and interquartile range with “Tukey” whiskers. WT = wild-type mice, 16p11.2 DEL = 16p11.2 deletion mice. Data are pooled for sex as no significant sex x genotype interaction was found (ANOVA). PPI was assessed at 3 different prepulse sound intensities –4, 8 and 16 dB above background. *n* = 16 (8 male) mice/genotype. **P < 0.01, ANOVA main effect of genotype.

While startle sensitivity was unaffected by the 16p11.2 deletion (Fig. 6a), there was a detriment in PPI in 16p11.2 DEL mice (Fig. 6b), supporting impaired pre-attentional processing in these animals. While the effect of the deletion on PPI appeared to be most evident at the lower prepulse intensities of 4 and 8 dB, we found no evidence for a significant interaction between genotype and prepulse intensity on PPI, supporting a deficit in PPI across all prepulse intensities.

**Fig. 7:**
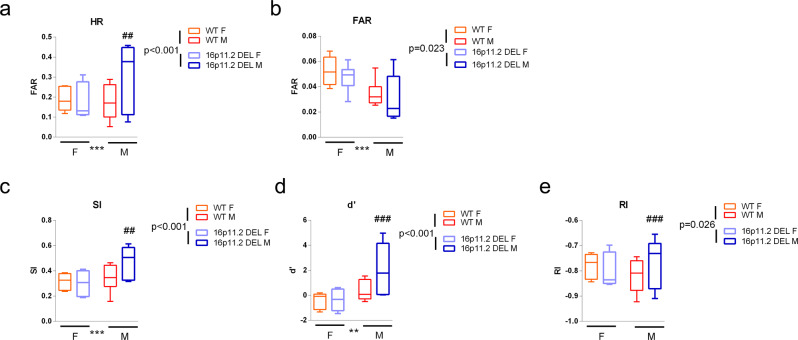
16p11.2 DEL mice have enhanced performance in the rCPT, when fully trained (stage 6), mainly due to a higher hit rate (HR) in 16p11.2 DEL males. **a** Hit Rate (HR), **b** False Alarm Rate (FAR), **c** Sensitivity Index (SI), **d** Perceptual Sensitivity (*d*’) and **e** Responsivity Index (RI) were monitored in 16p11.2 DEL mice and WT controls. Box plots show median and interquartile range with “Tukey” whiskers. *n* = 12 (6 male) mice/genotype. WT F = wild-type female mice, WT M = wild-type male mice, 16p11.2 DEL F = 16p11.2 deletion female mice, 16p11.2 DEL M = 16p11.2 deletion male mice. *p* values for overall genotype effects are shown (ANOVA). ^##^*p* < 0.01, ^###^*p* < 0.001 Tukey’s post hoc test compared to same sex WT group. **p < 0.01, ***p < 0.001 significant main sex effect.

The rCPT offers a translational opportunity to assess cognitive function in a paradigm close to that used clinically, and in which ASD individuals show altered performance^26^. Intriguingly, 16p11.2 DEL mice showed significantly better performance than WT mice during training for the rCPT. For example, over 12 sessions of stage 3 training, where animals are learning stimulus-specific responding and non-responding, the sensitivity index (SI) was significantly higher in 16p11.2 DEL mice (*p* = 0.016) (Supplementary Fig. 1). Similarly, over 10 sessions at stage 4 of training, where animals are faced with increasing complexity of non-reinforced stimuli, perceptual sensitivity (*d*’) was higher for 16p11.2 DEL as compared to WT mice, and the number of commission errors was lower (Supplementary Fig. 1). The stable performance of animals once the task was fully acquired (stage 6) is shown in Fig. 7. 16p11.2 DEL mice overall maintain enhanced performance as compared to WT mice when fully trained (Hit rate (HR), *p* = 0.001) (Fig. 7a–e). This effect was only evident in male 16p11.2 DEL mice (sex x genotype interaction *p* < 0.001, Fig. 7a). Males in general had a higher HR (*p* < 0.001), higher SI (*p* < 0.001) and higher *d*’ (*p* = 0.006) when compared to female mice, but 16p11.2 DEL males have higher performance in all these indices as compared to WT males (*p* < 0.001) (Fig. 7a, c, d).

**Fig. 8:**
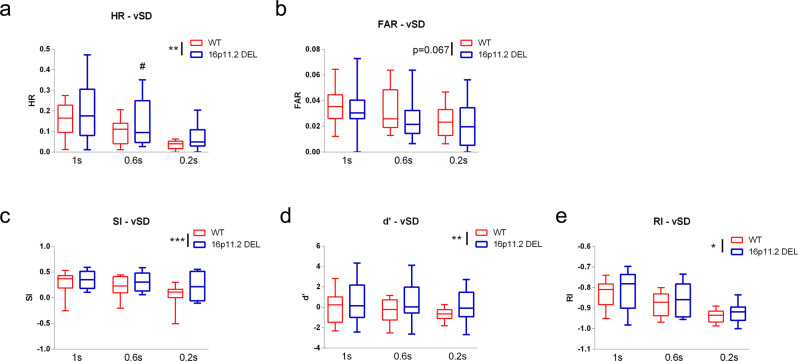
16p11.2 DEL mice have enhanced performance in the rCPT under conditions of increased attentional load (variable stimulus duration). **a** Hit rate (HR), **b** False Alarm Rate (FAR), **c** Sensitivity Index (SI), **d** Perceptual Sensitivity (*d*’) and **e** Responsivity Index (RI) were monitored in 16p11.2 DEL mice and WT controls under 3 different stimulus durations—1 s (as in the standard conditions at stage 6), Box plots show median and interquartile range with “Tukey” whiskers. 0.6 s and 0.2 s. *n* = 12 (6 male) mice/genotype. WT = wild-type, 16p11.2 DEL = 16p11.2 deletion mice. Data are pooled for sex. *p* values for overall genotype effects are as shown; *p < 0.05, **p < 0.01 (**a**) or *p* = 0.02 (**d**), *** *p* < 0.001, ANOVA; ^###^*p* < 0.001, Tukey’s post hoc test vs same stimulus WT group.

To further probe the enhanced attentional performance seen in 16p11.2 DEL mice we tested performance in the rCPT under conditions of increased attentional load, by reducing and varying the duration of stimulus presentation. As expected, HR and false alarm rate (FAR) decreased with shorter stimulus presentations (Fig. 8a, b). However, 16p11.2 DEL mice maintained their higher levels of performance compared to WT mice in all indices (Fig. 8a–e). Again, this mainly reflected the enhanced performance of 16p11.2 male mice (e.g. HR: genotype x sex interaction F(1,69) = 14.84, *p* < 0.001, DEL M > WT M Tukey *p* = 0.001).

Reaction times, for both correct and incorrect responses, were unaffected by 16p11.2 genotype, at any stage of the rCPT (Supplementary Table 2) indicating that there was no difference in speed of processing between the two genotypes.

## Discussion

Chromosomal microdeletions at 16p11.2 are one of the largest known risk factors for ASD and represent a unique opportunity for reverse translation. Here we report multi-scale ASD-relevant alterations in 16p11.2 DEL mice, including observations at the molecular, brain network and behavioural levels. The greater prevalence of sporadic ASD in males is also observed in 16p11.2 deletion carriers^11,44^. We, therefore, predicted that phenotypes would be more overt in male 16p11.2 DEL mice. Indeed, male-specific effects detected in 16p11.2 DEL mice were evident at all scales analysed. These observations provide integrative insight into the mechanisms by which 16p11.2 deletion increases the risk of developing ASD, and also provide translational measures against which putative therapeutics can be validated.

Increased E/I imbalance is a core feature of ASD^45–47^ and is also observed in relevant genetic mouse models^29,48^, with some behavioural deficits corrected by optogenetic modulation of E/I balance within the PFC^49^. Our findings of reduced *Pvalb* expression in the PFC and OFC, together with male-specific reductions in *Gad1* (septum, Ins and parietal cortices), support the putative loss of localised GABAergic inhibition in these regions. Interestingly, these molecular changes align with the increased neuronal excitability and decreased GABAergic neurotransmission reported in the PFC of 16p11.2 DUP mice^50^. Increased E/I balance may be present in 16p11.2 DEL mice, a suggestion supported by recent in vitro studies in the hippocampus^51^ and in vivo studies in cerebral cortex^48^ of these animals. Our data suggest that increased E/I balance in these animals reflects, in part, GABAergic interneuron dysfunction. However, evidence also supports enhanced glutamatergic neurotransmission in 16p11.2 DEL mice^51–53^. Notably, our GABA gene expression changes align with findings of reduced cortical GABA interneuron markers in ASD^30,31^. Decreased *GAD67* expression in ParC is reported in post-mortem tissue from autistic individuals (males), and is arguably the most robustly reported neurochemical phenotype. In addition, *Pvalb* expression in ParC is profoundly susceptible to developmental factors^54^, so the altered *Pvalb* expression seen here is suggestive of compromised neurodevelopmental processes in 16p11.2 DEL mice. One question that our study does not answer is whether the changes in GABAergic interneuron gene expression reflect changes in the number of interneurons in a given brain region, or altered gene expression levels alone. Either way, these changes are indicative of compromised GABAergic interneuron function. The cellular basis of these changes certainly warrants further systematic investigation, particularly given the observation that reduced numbers of Calb2-positive interneurons are seen in some cortical regions in 16p11.2 DEL mice^55^.

16p11.2 DEL mice show altered connectivity between the PFC and temporal cortical regions, and altered connectivity between these cortical regions and the septum/DB. Widespread dysconnectivity as a consequence of 16p11.2 deletion is supported, with altered connectivity in regions that contribute to a number of dysfunctional brain networks implicated in ASD, including the DMN, SalN, VAN and central executive network (CEN). These functional connectivity changes are accompanied by reduced GABAergic cell markers in cortical regions and the septum, consistent with the idea that locally altered E-I balance contributes to the altered inter-regional functional connectivity seen in these animals.

Human 16p11.2 DEL carriers also have compromised PFC-temporal-parietal connectivity^56^. In our data-driven analysis of large-scale brain network connectivity, we found reduced PFC connectivity in 16p11.2 DEL mice, including lost PFC hub connectivity (FrA) and lost PFC inter-regional connectivity with hippocampus, septum and nucleus accumbens (Figs. 1 and 2). Using rsFMRI in another 16p11.2 DEL mouse model, Bertero et al 2018^56^ also reported reduced long-range PFC connectivity. PFC hypoconnectivity is also evident in other mouse models relevant to the ASD including *Shank3* mutant^57^, *Cntnap* mutant^58^, *Nrxn1a* hemizygous^29^ and BTBR^59^ mice. However, in contrast to the previous report in 16p11.2 DEL mice, we also found PFC subfield-specific increases in connectivity, indicating complex PFC dysconnectivity in these animals. Observations of PFC dysconnectivity in ASD are also complex, with both increases and decreases reported^18,60^. Thus 16p11.2 DEL mice seem to replicate the functional complexity of PFC dysconnectivity seen in ASD, although further work is needed to map these connectivity parallels in detail. We also found profound dysconnectivity between the PFC and hippocampus in 16p11.2 DEL mice (Figs. 1 and 2), paralleling that seen in ASD^61^. As these functional connectivity changes include the dysconnectivity of regions within the DMN, this supports compromised DMN function in 16p11.2 DEL mice, consistent with compromised DMN connectivity in ASD ^17,62,63^ and human 16p11.2 DEL carriers^56^.

Fibres within the uncinate fasciculus (UF) link temporal regions (including the ventral hippocampus, AudC and PirC) with the frontal cortex (including PrL, IL, LO, DLO and Ins)^64,65^. One of the most robust structural abnormalities in ASD is compromised UF integrity^19,66–70^, and UF abnormalities are associated with impaired social and cognitive function^71,72^. UF abnormalities are also observed in humans with 16p11.2 deletions^73^. It is therefore intriguing that we observed functional dysconnectivity between these regions in 16p11.2 DEL mice (Figs. 1 and 2), which are accompanied by social deficits in these animals^74^. It will therefore be important for future work to characterise UF structure in 16p11.2 DEL mice, as a potentially valuable translatable biomarker.

We detected overt changes in cerebral metabolism in 16p11.2 DEL mice in the septum (MS), mammillary bodies (MB) and hippocampal subiculum (males only), and in PirC and AudC. The MB receives substantial afferent innervation from both subiculum and medial septum, so the data imply a particular impairment in this circuit. In fact, this is the precisely limbic circuitry where cellular morphology changes are reported in ASD^75,76^. In addition, decreased AudC activity is observed in individuals with level 1 (previously known as high-functioning) ASD^77^, paralleling our observations in 16p11.2 DEL mice. The male-specific elevation in subiculum activity identified here (Fig. 1) is also noteworthy, since ASD in males is associated with altered activity in this region^15,78^. The core limbic circuitry affected in male 16p11.2 DEL mice is summarised in Supplementary Fig. 2.

Substantial evidence supports Insular dysfunction in ASD, with decreased activity and hypoconnectivity supported^13,14,79^, including Insular-OFC hypoconnectivity in individuals with level 1 ASD^37^. This Insular dysfunction is paralleled in 16p11.2 DEL mice, where its hub status was lost along with connectivity to OFC, perirhinal cortex, nucleus accumbens and dorsolateral striatum. The Insular has key functions in interoreception, integrating external and internal stimuli and salience, in part through its actions on SalN connectivity and modulation of the DMN and CEN^80^. Insular dysconnectivity to SalN/CEN regions in 16p11.2 DEL mice is consistent with the behavioural changes seen in these animals, and the association of SalN dysconnectivity with ASD symptoms^81,82^. Impaired interoception is thought to underlie other deficits in ASD, including impaired theory of mind (ToM) and deficits in social communication^83^. Thus the social deficits reported in 16p11.2 DEL mice^74^ may also be related to this Insular dysconnectivity.

MS hyperactivity is accompanied by abnormal (gained) connectivity to amygdala nuclei and the mPFC (IL) in 16p11.2 DEL mice. By contrast, MS connectivity to other PFC/OFC subfields is lost, indicating a profound rearrangement of the functional connectivity of this region. In parallel GABAergic interneuron gene expression was reduced in the septum of 16p11.2 DEL mice, consistent with the increased metabolism in this area resulting from local disinhibition. Whilst anatomical evidence supports septum changes in ASD^76^, to our knowledge this is the first time that functional dysconnectivity of the septum with other brain regions has been identified in an ASD-relevant rodent model. Interestingly, we have recently reported reduced sociability in 16p11.2 DEL mice^74^. Since reduced GABAergic inhibition in the LS is sufficient to cause a loss of sociability in juvenile rats^84^, the reduced *Gad1* (*GAD67*) expression seen in the LS of male 16p11.2 DEL mice (Fig. 4) may relate to the social deficits reported in these animals, and may be of relevance to social deficits seen in ASD. MS forms part of a network important in affiliative behaviours, such as social connection. There are anatomical projections from MS to the IL, Sub, mammillary bodies and amygdala in rodents and humans^85–87^. Interestingly, the direct GABAergic projections from MS to the hippocampus, including the Sub^86,87^, inhibit subicular inhibitory interneurons, thereby disinhibiting subicular principal cells. Thus the compromised GABAergic gene expression seen in the MS of male 16p11.2 DEL mice may contribute to the subicular hyperactivity seen in these animals.

ASD individuals exhibit impaired PPI^88^, with the impairment diminishing to some extent with age. We observe a deficit in PPI in relatively young, 16p11.2 DEL mice. Other studies have reported no change in PPI in 16p11.2 DEL mice^89,90^. However, the mice in these earlier studies had a mixed genetic background. The mixed background may increase variability and decrease statistical power, potentially explaining the discrepancy. Two other studies have reported that PPI is dramatically decreased, to the point of being absent, in a different genetic strain of 16p11.2 DEL mice^52,91^. However, the strain used in these studies was deaf, so it is difficult to draw translationally-relevant conclusions from the results.

Mechanistically, a complex network involving AudC, PrL, ParC, Ins, TRN, septum, hippocampus, habenula and inferior colliculi is involved in PPI^39,92^, with the habenula in particular possessing strong functional connectivity with both AudC and Ins^40^. Considering the altered functional connectivity we observe in 16p11.2 DEL mice, and its striking overlap with this circuitry, it is no surprise to find that PPI is impaired. This includes, disrupted functional connectivity between the habenula and the AudC and Ins in 16p11.2 DEL mice (Figs. 1 and 2), along with disturbed PFC, septum and hippocampal connectivity. Moreover, GABAergic cell function is strongly implicated in the regulation of PPI, as evidenced by PPI deficits in *Pvalb* deficient mice^93^ and in mice with selective *Gad1* deficiency in Pvalb-expressing neurons^94^. Thus the PPI deficit is consistent with the GABAergic deficits seen in 16p11.2 DEL mice. The PPI deficit identified in 16p11.2 DEL mice represent a potentially translational phenotype, particularly when integrated with the functional neuroimaging and GABAergic cell deficits identified in these animals. Table 2 summarises the relationship between ASD-relevant phenotypes and brain region function in humans, and the overlap with the dysfunctional brain regions present in 16p11.2 DEL mice.

**Table 2:** Correspondence between human clinical studies and the present 16p11.2 DEL mouse preclinical study, in brain regions affected and linked to ASD-relevant behavioural phenotypes.

ASD-relevant behaviour	Regions involved, from human imaging	Citations	Regions affected in this 16p11.2 DEL mouse study
PPI deficits	Ins, PrL, AudC, ParC, TRN, MS, Hb, IC	^40^	Ins, PrL, AudC, ParC, TRN, MS, Hb
Altered attentional performance	Ins, PrL, OFC, cingulate, ParC, MS, Sub	^41,114^	Ins, PrL, OFC, ParC, MS, Sub, Accumbens
Impaired sociability^74^	Ins, OFC, amygdala	^37^	Ins, OFC

Attentional capabilities in ASD have attracted considerable interest, with debate over the attentional domains affected, including selective, sustained and shifting attention. Some aspects of attentional processing are known to be enhanced in autistic individuals^24,26,95^. In particular, autistic individuals can out-perform control subjects in tests involving visual and auditory search^2,24,95–97^, with debate over whether this enhanced performance relates to altered attentional or perceptual mechanisms^95^. Recent evidence suggests that enhanced perceptual capacity might underlie both superior performance in visual search and some of the deficits seen in other attentional abilities, for example through higher distractibility^24^. The improved attentional performance of 16p11.2 DEL mice in the visual rCPT task aligns with these human studies. This was largely due to enhanced performance in male 16p11.2 DEL mice, which is interesting given the greater prevalence of ASD in males.

Whilst not directly comparable to the present data, the performance of 16p11.2 DEL mice has been assessed in other cognitive tasks. 16p11.2 DEL mice are slower in acquiring competency in a pairwise discrimination and reversal learning touchscreen task^91^. The difference between the results in this task and our rCPT results may reflect the differing cognitive domains involved (visual discrimination and intradimensional flexibility versus visual discrimination and attention), or the fact that the mice in the other study had a mixed (C57Bl6/129/CD-1) genetic background. The mice on the mixed genetic background also show a higher neonatal fatality rate than those used in our study, further suggestive of other genetic factors interacting with the 16p11.2 deletion. In addition, these mixed background mice, being deaf, also lacked the auditory feedback that accompanies correct responses in these touchscreen tests, which may make learning more difficult.

In our study, enhanced rCPT performance in 16p11.2 DEL mice was observed during training, when fully trained, and when the attentional demands of the task were acutely increased. From a translational perspective, and consistent with these observations, ASD individuals (predominantly male) show increased *d*’ in the CPT, especially under conditions of increased attentional load^24^. This supports the translational relevance of the 16p11.2 DEL mouse model in this regard. In terms of the relevant neurocircuitry, it is intriguing that Ins connectivity to components of the SalN (nucleus accumbens) is altered in 16p11.2 DEL mice (Fig. 2), as the Ins influences interactions between the DMN (“off task”) and CEN/SalN (“on task”) networks^41,98^. Interestingly, fMRI studies in ASD support decreased inactivation of the DMN during cognitive tasks, which may relate to Ins dysconnectivity^62^. For optimal cognitive performance, a reciprocal antagonistic relationship is required between these networks^99^. The DMN (e.g. mPFC and posterior cingulate) is typically deactivated during attention-demanding tasks, whereas the CEN (particularly the Dorsal Attention Network comprising frontoparietal regions) is activated. In ASD there are deficits in the DMN, with both hyper- and hypo-connectivity profiles reported^17^. The improved rCPT performance seen in 16p11.2 DEL mice may result from a shift in the reciprocal antagonistic relationship between the DMN and CEN, putatively supported by our data showing dysfunction in DMN and CEN regions. This certainly warrants further investigation.

Some genetic and toxicological mouse models of aspects of ASD show deficits in simple tests of cognitive function (e.g. novel object recognition, delayed match to position, fear conditioning, Morris water maze). While the translational relevance of some of these tests is in question, overt impairment has generally been observed, particularly with manipulations associated with more severe ends of the spectrum^100–104^. There is a relative lack of data from more sophisticated and translational cognitive tests. However, in models of genetic lesions associated with more severe impairment, including a mouse model of Fragile X syndrome^105^ and in mice of the inbred BTBR strain which lacks a corpus callosum^106^, attentional deficits have been observed. However, enhanced cognitive performance is detected in other mouse models of ASD with high construct validity. For example, enhanced learning is seen in various mouse models with ASD-related mutations, including *Shank1*^−/−^^107^,*Taok2*^−/−^^108^, *Ctnbb1*^−/–^ (Pvalb cell conditional)^109^, *Nrxn1α +/−* mice^110^ and also in *Aspm*^−/−^ mice that show reduced TRN Pvalb expression^111^. A recent publication reports that mice with an ASD-associated neuroligin-3 mutation also show enhanced ability in the rCPT^112^. Thus an emerging picture of enhanced performance in specific cognitive domains is evident in mouse models relevant to ASD.

We note that hypofunction in the ERK pathway is associated with cognitive impairment rather than enhancement^113^. However, mice with *Taok2* deletion exhibit clearly enhanced performance compared to WTs in a spatial learning task^108^. Hence the decreased *Taok2* gene dosage present in 16p11.2 DEL mice may contribute to the cognitive phenotypes observed in the current study.

Interestingly, increased visual search efficiency in ASD is associated with heightened activation of ParC^114^. As increased perceptual load, where altered attentional performance in autistic individuals is most evident (Remington 2012), is associated with greater ParC recruitment^115^, the decreased GABAergic gene expression seen in ParC in 16p11.2 DEL mice may result in elevated local E/I balance which, in turn, may contribute to enhanced attentional performance in these animals.

We have recently reported an extensive characterisation of CNS dysfunction in mice with the corresponding duplication (16p11.2 DUP mice)^116^. Since this strain is on the same genetic background, it is informative to compare the two studies. The changes in brain metabolism and functional connectivity seen are more marked in 16p11.2 DEL as compared to 16p11.2 DUP mice. This could have been predicted—considering the 50% change in gene dosage in 16p11.2 DEL as compared to the 33% change in 16p11.2 DUP mice. Interestingly, both 16p11.2 DEL and 16p11.2 DUP mice show impaired OFC connectivity. For 16p11.2 DUP mice this is most pronounced in terms of connectivity to hippocampus and amygdala^116^, whereas for 16p11.2 DEL mice this is most pronounced for connectivity to the hippocampus and Ins. Why the OFC-hippocampal axis should be especially sensitive to abnormal 16p11.2 gene dosage is unclear, but the data focus attention on this interaction as potentially key for the aetiology of 16p11.2-related neurodevelopmental disorders.

PPI is impaired in both 16p11.2 DEL (Fig. 5) and 16p11.2 DUP mice^116^. Thus either elevated or reduced gene dosage in this region impairs pre-attentional sensorimotor gating. This is consistent with the fact that both patients with schizophrenia, where risk is associated with 16p11.2 duplication, and autistic individuals, where risk is associated with 16p11.2 deletion, show compromised PPI^88^. It is also consistent with the observation that both the deletion and duplication impact on neurocircuitry that contributes to PPI in mice.

In terms of attentional processing, we have detected reciprocal phenotypes for the reciprocal CNVs. While 16p11.2 DUP mice show impaired rCPT performance^116^, 16p11.2 DEL mice show enhanced rCPT performance (Figs. 7 and 8). This is similar to humans, where individuals carrying the duplication show more impaired working memory and executive function than those carrying the deletion^9^. Future work dedicated to directly comparing both duplication and deletion-carrying individuals in the human CPT would therefore be of great translational interest.

In this work, we have undertaken a multi-scale, integrative characterisation of mice carrying the 16p11.2 deletion to elucidate the translational alignment of this mouse model with observations in ASD, and to further identify the mechanisms through which the 16p11.2 deletion increases the risk of developing neurodevelopmental disorders. Key observations include translationally aligned disruptions in GABAergic gene expression, putatively contributing to locally altered E-I balance. In addition, we identify perturbed regional metabolism and functional connectivity that aligns with the impaired pre-attentional sensorimotor gating and superior attentional performance seen in these animals. These findings align with those reported in autistic individuals. Thus we have established the translational utility of the 16p11.2 DEL mouse model for ASD and highlighted a range of phenotypes against which the validity of future therapeutics can be tested.

## Methods

### Ethics approval and consent to participate

The study was conducted under UK Home Office regulations, and was approved by the University of Glasgow College of Medical, Veterinary and Life Sciences Ethics Committee.

### Animals

Male and female mice (Jackson Laboratory, Stock No. 013128) hemizygous for the 0.44 Mb region of mouse chromosome 7, syntenic to the human 16p11.2 deletion, were generated by Mills and colleagues^117^. Mice were backcrossed onto the C57BL/6 N background using “Max-Bax” SNP-guided back-crossing (Charles River) to generate experimental mice, so that, in contrast to some other studies using this strain, the mice for this study are effectively congenic^74^. Primers used for genotyping are listed in Supplementary Table 4. Genotpyping was performed using a PCR protocol of 94 °C for 2 min, then 10 cycles of 94 °C for 20 s, 65 °C for 15 s and 68 °C for 10 s, followed by 28 cycles of 94 °C for 15 s, 60 °C for 15 s and 72 °C for 10 s. 16p11.2 DEL mice, and littermate wild-type (WT) controls, were group-housed, with food and water ad libitum. All work was approved by the University of Strathclyde and Lancaster University Animal Welfare and Ethics Review Boards (AWERB) and conducted in accordance with the Home Office (UK) Animals (Scientific Procedures) Act 1986.

### ^14^C-2-deoxyglucose functional brain imaging

We used our standard protocols^27,28^ for ^14^C-2-deoxyglucose (^14^C-2-DG) metabolic functional brain imaging in mice aged 10–11 weeks old. Autoradiographic signal intensity was measured using the MCID system, by an operator blind to the experimental identity of the samples. The group sizes: WT, *n* = 12 (6 male) and 16p11.2 DEL, *n* = 12 (6 male) mice, based on our previous experiments with GM mice, were estimated to provide 85% power to detect an effect at *p* < 0.05 (Minitab).

Network science algorithms, using the igraph package^118^ in R^119^, were used to determine functional brain network structure, as described previously^27,28,116,120^. These algorithms permit the assessment of regional importance and connectivity in the context of the whole brain network, with highly connected hub brain regions being identified as having high centrality. In this way, we determined altered hub brain region status on the basis of both degree (k_i_) and betweenness (B_c_) centrality. The significance of group differences in centrality were determined by permutation (55,000 permutations), with significance set at *p* < 0.05.

PLSR analysis allows the determination of the inter-regional connectivity for a selected seed brain region, thus allowing the characterisation of the inter-regional connectivity changes that underlie the altered functional hub status of a brain region. Data were analysed with the Pls package^121^ in R, according to our published procedures^27–29^. If the lower bound of the 95% confidence interval (CI) of the variable importance to the projection (VIP) statistic (estimated by jack-knifing) exceeded 1.0, then significant inter-regional connectivity to the seed region was considered to exist within the experimental group. Significantly altered connectivity in 16p11.2 DEL mice was determined by comparison of the VIP statistic via calculation of the standardised *z* score, with a *z* score >1.96 or <−1.96 considered to be significant. Data are reported for two levels of significance, *z* = 1.96 (equivalent to *p* = 0.05) and *z* = 2.58 (equivalent to *p* = 0.01). Significantly altered connectivity was confirmed by a 95% CI of the VIP > 1.0 in one experimental group, but not the other (95% CI lower bound VIP < 1.0).

### In situ hybridisation (ISH)

In situ hybridisation (ISH) was used to assess regional mRNA expression according to our standard protocol^116,122,123^ using ^35^S-labelled 45mer oligonucleotide probes. Sequences of oligonucleotide probes used are listed in Supplementary Table 4. Brains (from mice 9–11 weeks of age) were sectioned and mounted onto slides in same sex, different genotype pairs. 20 μm frozen cryostat sections, mounted on microscope slides coated with poly-L-Lysine (Sigma-Aldrich, UK), were fixed for 10 min in 4% paraformaldehyde (Sigma-Aldrich, UK) on ice. Hybridisation between sections and oligonucleotides—3’end-labelled with ^35^S-dATP and terminal deoxynucleotidyl transferase (ThermoFisher, UK)—was performed at 42 °C overnight in a humidified chamber. All samples were processed at the same time, for each target mRNA. Sections were then washed in standard saline citrate buffer (Sigma-Aldrich, UK) for 1 h at 55 °C, before being dehydrated in ethanol. Specificity of labelling was monitored using competition controls (25× excess of unlabelled oligonucleotide)^122,124,125^, and ImageJ was used to assess autoradiographic signal intensity, by a researcher blind to the identity of the samples. Eight sections per animal were analysed. Each microscope slide contained sections from one WT and one 16p11.2 DEL mouse of the same sex. The signal intensity for each region was measured on both sides of the brain in each section. Similarly, background signal values were taken from adjacent areas lacking specific signal (as assessed by the competition control) on both sides of the brain in each section. These were then subtracted from the signal in the areas with specific labelling. Both resulting “specific” signal values were included in the statistical analysis, and “side” (left vs right hemisphere) included as a random factor. We found no evidence that brain hemisphere significantly impacted on gene expression or influenced the impact genotype on the expressions of the gene targets analysed. The group sizes: WT, *n* = 12–13 (8–9 male) and 16p11.2 DEL, *n* = 12 (8 male) mice, were estimated, based on our previous experience, to give 99% power to detect an effect at *p* < 0.05 (Minitab).

### Prepulse inhibition (PPI)

Mice were 13–14 weeks of age, and SR-LAB chambers (San Diego Instruments, San Diego, CA) were used as previously described^116,126^. Each chamber consisted of a sound attenuated cabinet (inside height: 28.7 cm, inside width: 28.7 cm, inside depth: 30 cm) that was lit and ventilated, with a Plexiglass cylinder (3.7 cm inner diameter, 12.7 cm long) situated on top of a removable stand in which a piezoelectric accelerometer was attached underneath. An initial startle curve (random presentation of 60 trials of 65 dB, 69 dB, 73 dB, 77 dB, 85 dB, 90 dB, 100 dB, 110 dB, 120 dB—full spectrum white noise) was obtained. For PPI determination, mice were allowed to acclimatise to the 65 dB background noise for 5 min, and 5 × 40 ms, 120 dB startling stimuli were then played to partially habituate the animals to the startling stimulus. Mice were then tested over 60 trials. The session consisted of 10 repetitions of each of the following prepulse trials: a 20 ms prepulse of either 4, 6, or 8 dB above background, followed by a 100 ms inter-pulse interval, then a 40 ms startling stimulus at 120 dB above background. Randomly interspersed between prepulse trials were 10 × 120 dB startling stimuli alone and 10 x “no stimulus” trials in which movements were recorded but no stimulus was delivered. These trials were presented in random order with inter-trial intervals averaging ~15 s, but were either (randomly) 12, 13, 14, 15, 16, or 17 s long. Movements of the animal were recorded for 40 ms from the beginning of the 120 dB startling stimulus, or, in the case of “no stimulus” trials, from the end of the inter-trial interval for 10 ms. The session finished with 5 × “120 dB startle only” trials to give an indication of overall habituation to the startle response the mice exhibited when compared with the first 5 × “120 dB startle only” trials. % PPI was calculated in relation to startle reactivity at 120 dB (Eq. 1).

1 $${{{{{\rm{PPI}}}}}}=\, 	({{{{{\rm{startle}}}}}}\,{{{{{\rm{reactivity}}}}}}\,{{{{{\rm{at}}}}}}\,120\,{{{{{\rm{dB}}}}}}\,{{{{{\rm{without}}}}}}\,{{{{{\rm{prepulse}}}}}} - {{{{{\rm{startle}}}}}}\,{{{{{\rm{reactivity}}}}}}\,{{{{{\rm{at}}}}}}\,120\,{{{{{\rm{dB}}}}}}\,{{{{{\rm{with}}}}}}\,{{{{{\rm{prepulse}}}}}}) \\ 	 \, \times 100/{{{{{\rm{startle}}}}}}\,{{{{{\rm{reactivity}}}}}}\,{{{{{\rm{at}}}}}}\,120\,{{{{{\rm{dB}}}}}}\,{{{{{\rm{without}}}}}}\,{{{{{\rm{prepulse}}}}}}.$$

The group sizes: WT, *n* = 16 (8 male) and 16p11.2 DEL, *n* = 16 (8 male) mice, according to our previous experience with this test in GM mice, were estimated to provide 92% power to detect an effect at *p* < 0.05 (Minitab).

### Rodent continuous performance test (rCPT)

The rCPT was conducted using Campden instruments mouse touchscreen operant boxes and ABETII touch software. Mice (11–13 weeks of age) were trained according to Kim et al.,^127^ and as previously described^116^. Mice were assessed for attentional performance (hit rate (HR) and false alarm rate (FAR)), along with composite measures of performance analogous to those used clinically (Sensitivity index—SI, perceptual sensitivity—*d*’ and index of response bias—RI), and measures of processing speed (reaction time).

The task stimuli were presented in the centre panel of three discrete sections of the touchscreen, accessible via a 3 aperture horizontal mask. Correct responses were rewarded with (YazooTM) strawberry milkshake (70 µl), along with an auditory stimulus and illumination of the food reward hopper. Stimuli were either rewarded (S+) or punished (S−) by a correction phase, involving repeated stimulus presentation until the response is correctly withheld. Successive stages of basal response learning (stage 1), stimulus-specific responding (stage 2) and stimulus-specific responding and non-responding (stage 3), were followed by manipulations that increases cognitive load, including an increased number of S- stimuli and reduced stimulus presentation times (2 sec, 1.5 sec, 1 sec; limited hold 2.5 sec, 2 sec, 1.5 sec) in stages 4–6 of the task. The group sizes: WT, *n* = 12 (6 male) and 16p11.2 DEL, *n* = 12 (6 male) mice, according to our previous experience with GM mice in this task, were estimated to provide 86% power to detect an effect at *p* < 0.05 (Minitab).

The parameters calculated were: Hit Rate (HR)—the rate at which animals respond to the correct (S+) stimulus (Eq. 2). False Alarm Rate (FAR)—the rate at which animals respond to the incorrect (S−) stimulus (Eq. 3); Sensitivity Index (SI)—the perceptual discriminability between the correct (S+) and incorrect (S−) stimuli, with higher values indicate better discrimination;^127^ Perceptual Sensitivity (*d*’) (Eq. 4); Responsivity Index (RI)—the criterion or willingness to make responses, with conservative responding indicated by low RI values and liberal responding indicated by high RI values^127^, see erratum for correct formula. Note that the LnBeta index, which is also widely used, and where low values indicate more liberal responding strategies, is the inverse of this measure.

2 $${{{{{\rm{HR}}}}}}={{{{{\rm{Hit}}}}}}/({{{{{\rm{Hit}}}}}}+{{{{{\rm{Miss}}}}}});$$

3 $${{{{{\rm{FAR}}}}}}={{{{{\rm{False}}}}}}\,{{{{{\rm{alarm}}}}}}/({{{{{\rm{False}}}}}}\,{{{{{\rm{alarm}}}}}}+{{{{{\rm{Correct}}}}}}\,{{{{{\rm{rejection}}}}}})$$

4 $${{{{{\rm{d}}}}}}\hbox{'}=\, 	{{{{{\rm{z}}}}}}({{{{{\rm{HR}}}}}})-{{{{{\rm{z}}}}}}({{{{{\rm{FAR}}}}}}),{{{{{\rm{where}}}}}}\,{{{{{\rm{z}}}}}}\,{{{{{\rm{scores}}}}}}\,{{{{{\rm{are}}}}}}\,{{{{{\rm{calculated}}}}}}\,{{{{{\rm{based}}}}}}\,{{{{{\rm{on}}}}}}\,{{{{{\rm{performance}}}}}}\\ 	 \, {{{{{\rm{of}}}}}}\,{{{{{\rm{WT}}}}}}\,{{{{{\rm{mice}}}}}}\,{{{{{\rm{under}}}}}}\,{{{{{\rm{standard}}}}}}\,{{{{{\rm{conditons}}}}}};$$

### Statistics and reproducibility

Analysis was performed blind to the experimental group wherever possible, as described above. Mice were allocated to test groups in randomised order. The use and analysis of technical replicates in the in situ hybridisation studies is described above. Group sizes in all studies were determined based on power analysis (>0.8, *p* < 0.05) using Minitab. Statistical significance was generally assessed by ANOVA (Minitab/R), with prior Box-Cox normalisation where data deviated substantially from normality. Post hoc comparisons were made using Tukey’s HSD or Mann-Whitney tests for specific planned comparisons between groups of particular interest. For ISH data analysis, 3-way ANOVA was employed (factors: genotype, sex and slide pairing). Details of statistical outputs are provided in Supplementary Table 1.

### Reporting summary

Further information on research design is available in the Nature Portfolio Reporting Summary linked to this article.

## Supplementary information

Supplementary Information

Reporting Summary

## Data Availability

The 16p11.2 DEL mice used in this study are available from Jackson laboratories (strain 013128). Data shown in the figures are deposited at Dryad: 10.5061/dryad.x69p8czp7.
