# Supplementary material for: 16p11.2 deletion mice exhibit compromised fronto-temporal connectivity, GABAergic dysfunction, and enhanced attentional ability
Source: Commun Biol. 2023 May 24;6:557. doi: 10.1038/s42003-023-04891-2 (PMC10209099; doi:10.1038/s42003-023-04891-2)

Supplementary Information –

Openshaw et al.

16p11.2 deletion mice exhibit compromised fronto-temporal connectivity, GABAergic dysfunction, and enhanced attentional ability

Supplementary Table 1: Statistical analysis details for Figures

Supplementary Table 2: Reaction times in the rCPT

Supplementary Table 3: Brain region abbreviations

Supplementary Table 4: Oligonucleotide sequences

Supplementary Figure 1: d’ data at stages 3 and 4 of training for rCPT, and no. of mistakes at stage 4.

Supplementary Figure 2: Summary of the core limbic circuitry affected in male 16p11.2DEL mice.

Supplementary Table 1 - Statistical analysis details					
**Figure**	**Paradigm**	**WT vs. 16p11 DUP (Effect of Genotype)**	**Genotype x sex**	**Effect of task stage**	**Effect of sex**
1	2DG MS	**$F_{(1,19)}$=13.64, p=0.002**	$F_{(1,19)}$=0.33, p=0.574		$F_{(1,19)}$=0.22, p=0.647
1	2DG Piri	**$F_{(1,19)}$=8.49, p=0.009**	$F_{(1,19)}$=0.47, p=0.500		$F_{(1,19)}$=1.08, p=0.312
1	2DG AudC	**$F_{(1,19)}$=7.34, p=0.014**	$F_{(1,19)}$=0.78, p=0.389		$F_{(1,19)}$=0.01, p=0.977
1	2DG MB	**$F_{(1,20)}$=5.81, p=0.026**	$F_{(1,20)}$=0.03, p=0.873		$F_{(1,20)}$=3.61, p=0.072
1	2DG Sub	$F_{(1,19)}$=1.97, p=0.177	**$F_{(1,19)}$=11.43, p=0.003**		**$F_{(1,19)}$=10.31, p=0.005**
3	Pvalb PrL	**$F_{(1,41)}$=33.69, p<0.001**	$F_{(1,41)}$=4.33, p=0.050		$F_{(1,41)}$=59.24, p<0.001
3	Pvalb IL	**$F_{(1,41)}$=26.04 p<0.001**	$F_{(1,41)}$=0.21, p=0.650		$F_{(1,41)}$=45.41, p<0.001
3	Pvalb MO	$F_{(1,41)}$=6.02, p=0.023	$F_{(1,41)}$=0.69, p=0.416		$F_{(1,41)}$=0.81, p=0.378
3	Pvalb LO	$F_{(1,41)}$=1.49, p=0.236	$F_{(1,41)}$=0.21, p=0.650		$F_{(1,41)}$=0.86, p=0.365
3	Calb PrL	**$F_{(1,47)}$=18.07, p<0.001**	**$F_{(1,47)}$=8.17, p=0.009**		$F_{(1,47)}$=0.24, p=0.628
3	Calb IL	**$F_{(1,47)}$=24.05, p<0.001**	**$F_{(1,47)}$=34.59 p<0.001**		$F_{(1,47)}$=2.54, p=0.124
3	Calb MO	$F_{(1,47)}$=0.04, p=0.835	**$F_{(1,47)}$=11.72, p=0.002**		$F_{(1,47)}$=4.72, p=0.040
3	Calb LO	$F_{(1,47)}$=2.02, p=0.168	$F_{(1,47)}$=5.67, p=0.026		$F_{(1,47)}$=5.65, p=0.026
3	GAD1 PrL	$F_{(1,45)}$=0.03, p=0.854	$F_{(1,45)}$=5.35, p=0.027		$F_{(1,45)}$=0.04, p=0.839
3	GAD1 IL	$F_{(1,45)}$=0.37, p=0.547	$F_{(1,45)}$=2.20, p=0.148		$F_{(1,45)}$=1.53, p=0.226
4	Pvalb MS	$F_{(1,47)}$=2.58, p=0.117	$F_{(1,47)}$=5.67, p=0.023		$F_{(1,47)}$=3.29, p=0.078
4	Pvalb LS	$F_{(1,45)}$=0.94, p=0.339	$F_{(1,45)}$=0.10, p=0.759		$F_{(1,45)}$=095, p=0.338
4	Calret MS	$F_{(1,51)}$=2.85, p=0.100	$F_{(1,51)}$=1.60, p=0.213		$F_{(1,51)}$=1.09, p=0.304
4	Calret LS	$F_{(1,51)}$=0.00, p=0.956	$F_{(1,51)}$=0.06, p=0.813		$F_{(1,51)}$=0.42, p=0.520
4	GAD1 MS	$F_{(1,51)}$=3.87, p=0.057	$F_{(1,51)}$=2.00, p=0.166		$F_{(1,51)}$=2.84, p=0.100
4	GAD1 LS	$F_{(1,51)}$=1.42, p=0.241	**$F_{(1,51)}$=7.62, p=0.009**		$F_{(1,51)}$=1.15, p=0.290
4	SST INS	$F_{(1,47)}$=0.80, p=0.379	$F_{(1,47)}$=1.04, p=0.316		$F_{(1,47)}$=1.94, p=0.173
4	Pvalb INS	$F_{(1,45)}$=0.94, p=0.339	$F_{(1,45)}$=0.10, p=0.759		$F_{(1,45)}$=095, p=0.338
4	GAD1 INS	$F_{(1,47)}$=0.03, p=0.859	$F_{(1,47)}$=5.44, p=0.026		$F_{(1,47)}$=0.01, p=0.916
5	Pvalb CA1	$F_{(1,88)}$=9.08, p=0.004	$F_{(1,88)}$=0.83, p=0.365		$F_{(1,88)}$=4.30, p=0.042
5	Calb CA1	$F_{(1,92)}$=0.50, p=0.483	$F_{(1,92)}$=3.56, p=0.063		$F_{(1,92)}$=8.82, p=0.004
5	SST TRN	**$F_{(1,70)}$=7.32, p=0.009**	$F_{(1,70)}$=0.75, p=0.392		$F_{(1,70)}$=11.24, p=0.002
5	Pvalb TRN	$F_{(1,81)}$=2.17, p=0.147	$F_{(1,81)}$=1.21, p=0.277		$F_{(1,81)}$=0.02, p=0.901
5	GAD1 TRN	$F_{(1,98)}$=3.50, p=0.066	$F_{(1,98)}$=2.61, p=0.110		$F_{(1,98)}$=1.35, p=0.250
5	Pvalb ParCx	$F_{(1,51)}$=17.22, p<0.001	$F_{(1,51)}$=4.56, p=0.041		$F_{(1,51)}$=3.82, p=0.062
5	GAD1 ParCx	$F_{(1,47)}$=0.06, p=0.815	$F_{(1,47)}$=4.56, p=0.040		$F_{(1,47)}$=0.13, p=0.722
	GAD1 PirCx	$F_{(1,51)}$=1.25, p=0.27	$F_{(1,51)}$=0.06, p=0.80		$F_{(1,51)}$=0.52, p=0.48

6	Startle			$F_{(9,252)}$ = 84.40 p = 0.0001	
6	PPI (%)	**$F_{(1,95)}$=10.38, p=0.002**	$F_{(1,95)}$=0.15, p=0.70	$F_{(1,95)}$=39.57, p<0.001	
		CPT			
7	Stage 6 HR	**$F_{(1,263)}$=46.95, p<0.001**	$F_{(1,263)}$=75.87, p<0.001 DEL M > WT M Tukey $F_{(1,263)}$=46.75, p<0.001 DEL M > WT M Tukey		$F_{(1,263)}$=44.36, p<0.001
7	Stage 6 FAR	$F_{(1,263)}$=5.22, p=0.023	ns		$F_{(1,263)}$=77.08, p<0.001
7	Stage 6 SI	**$F_{(1,263)}$=31.20, p<0.001**	$F_{(1,263)}$=46.75, p<0.001 DEL M > WT M Tukey 0.01		$F_{(1,263)}$=99.31, p<0.001
7	Stage 6 RI	$F_{(1,263)}$=5.03, p=0.026	$F_{(1,263)}$=31.92, p<0.001 DEL M > WT M Tukey 0.001		ns
7	Stage 6 d'	$F_{(1,259)}$=47.29, p<0.001	$F_{(1,259)}$=39.56, p<0.001 WT M > WT F		$F_{(1,259)}$=157.8, p<0.001 M > F
8	VSD HR	**$F_{(1,69)}$=11.41, p=0.002**	**$F_{(1,69)}$=14.84, p<0.001**	$F_{(1,69)}$=39.21, p<0.001	$F_{(1,69)}$=6.14, p=0.018
8	VSD FAR	$F_{(1,69)}$=3.56, p=0.067	$F_{(1,69)}$=0.42, ns	$F_{(1,69)}$=4.47, p=0.018	$F_{(1,69)}$=9.20, p=0.004
8	VSD SI	**$F_{(1,63)}$=31.82, p<0.001**	$F_{(1,63)}$=9.41, p=0.004 DEL M > WT M Tukey 0.001	ns	ns
8	VSD RI	$F_{(1,71)}$=4.55, p=0.039	$F_{(1,71)}$= 7.98, p=0.007	$F_{(1,71)}$=59.79, p<0.001	ns
8	VSD d'	$F_{(1,71)}$=13.39, p=0.001	$F_{(1,71)}$=11.07, p=0.002 DEL M > WT M Tukey 0.001	$F_{(1,71)}$=36.64, p<0.001	$F_{(1,71)}$=8.10, p=0.007

Supplementary Table 2: Reaction times in the rCPT (s)

**Stage of task**	**WT females**	**WT males**	**DEL females**	**DEL males**
Stage 6				
Correct Choice	0.74 +/- 0.03	0.74 +/- 0.05	0.79 +/- 0.02	0.76 +/- 0.04
Incorrect Choice	0.73 +/- 0.02	0.71 +/- 0.02	0.68 +/- 0.04	0.64 +/- 0.08
Reward collection	1.82 +/- 0.27	1.30 +/- 0.15	1.86 +/- 0.21	1.35 +/- 0.20
Distractor				
Correct Choice	1.04 +/- 0.09	0.98 +/- 0.10	0.86 +/- 0.14	0.73 +/- 0.18
Incorrect Choice	1.15 +/- 0.05	1.22 +/- 0.10	1.08 +/- 0.04	1.13 +/- 0.11
Reward collection	2.10 +/- 0.32	1.31 +/- 0.14	1.66 +/- 0.19	0.98 +/- 0.21
vSD				
Correct Choice	0.64 +/- 0.02	0.60 +/- 0.03	0.66 +/- 0.02	0.63 +/- 0.04
Incorrect Choice	0.56 +/- 0.04	0.49 +/- 0.03	0.50 +/- 0.02	0.44 +/- 0.07
Reward collection	2.03 +/- 0.39	1.37 +/- 0.18	1.44 +/- 0.18	1.21 +/- 0.14

Supplementary Table 3: Brain region abbreviations

Prefrontal Cortex	***Mesolimbic***	***Olfactory***
Anterior Prelimbic Cortex (aPrL)	Nucleus Accumbens Core (AcbC)	Olfactory Bulb (OB)
Frontal Association Area (FRA)	Nucleus Accumbens Shell (AcbSh)	***Neuromodulatory***
Dorsolateral Orbital Cortex (DLO)	Ventral Tegmental Area (VTA)	Dorsal Raphe (DR)
Medial Orbital Cortex (MO)	***Basal Ganglia***	Median Raphe (MR)
Lateral Orbital Cortex (LO)	Dorsolateral Striatum (DLST)	***Hippocampus***
Medial Prelimbic Cortex (mPrL)	Ventromedial Striatum (VMST)	Dorsal Subiculum (DSub)
Infralimbic Cortex (IL)	Globus Pallidus (GP)	Dorsal Cornu Ammonis 1 (DH CA1)
Cingulate Cortex (Cg1)	Substantia Nigra pars Compacta (SNC)	Dorsal Cornu Ammonis 2 (DH CA2)
Cortex	Substantia Nigra pars Reticulata (SNR)	Dorsal Molecular Layer (DH Mol)
Insular Cortex (Ins)	***Amygdala***	Dorsal Dentate Gyrus (DH DG)
Piriform Cortex (Piri)	Basolateral Amygdala (BLA)	Ventral Cornu Ammonis 1 (VH CA1)
Retrosplenial Cortex (RSC)	Central Amygdala (CeA)	Ventral Cornu Ammonis 3 (VH CA3)
Somatosensory Cortex (S1)	Medial Amygdala (MeA)	Ventral Molecular Layer (VH Mol)
Thalamus	***Auditory***	Ventral Dentate Gyrus (VH DG)
Anteromedial Thalamus (AM)	Inferior Colliculus (IC)	Entorhinal Cortex (ENT)
Thalamic Reticular Nucleus (TRN)	Medial Geniculate (MG)	Perirhinal Cortex (PRh)
Anterior Reticular Thalamus (aRT)	**Septum/Diagonal Band of Broca (DB)**	***Multimodal***
Anteroventral Thalamus (AV)	Lateral Septum (LS)	Habenula (Hab)
Mediodorsal Thalamus (MD)	Medial Septum (MS)	Mamillary Body (MB)
Ventrolateral Thalamus (VL)	Horizontal Limb of DB (HDB)	Corpus Callosum (CC)
Ventromedial Thalamus (VM)	Vertical Limb of DB (VDB)	

Supplementary Table 4: Oligonucleotide sequences

Gene	Methodology	Sequence
Pvalb	In situ hybridisation	AGTGGAGAATTCTTCAACCCCAATCTTGCCGTCCCCATCCTTGTC
Calb1	In situ hybridisation	AATTCCTATTTTTCCATCATCTCTCTGTCCATATTGATCCACAAA
Calb2	In situ hybridisation	CTGTTGGATGTTCATCTCCTTCTTGTTCTTCTCATACAGATCCTT
SST	In situ hybridisation	CAAATCCTCGGGCTCCAGGGCATCATTCTCTGTCTGGTTGGGCTC
Gad1	In situ hybridisation	CCTGCACACATCTGGTTGCATCCTTGGAGTATACCCTTTTCCTTG
16p11 wt	Genotyping (fwd)	TACCTCACACCTCAGTTCCAAGTGG
16p11 wt	Genotyping (rev)	GGGTGGGAGTTCTCGCCTATCACAG
16p11 mutant	Genotyping (fwd)	GGTAGAATTTCGAGGTCGCTAG
16p11 mutant	Genotyping (rev)	CAA GCT GAT CCG GAA CCC

Supplementary Figure 1: d' data at stages 3 and 4 of training for rCPT, plus no. of mistakes at stage 4. Data shown as mean +/-standard error of the mean

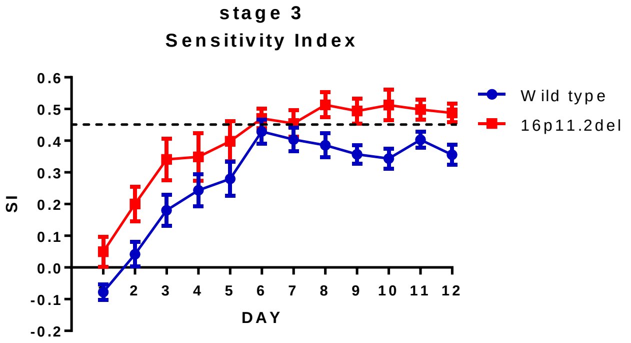

ANOVA: Main effect of genotype (p = 0.016).

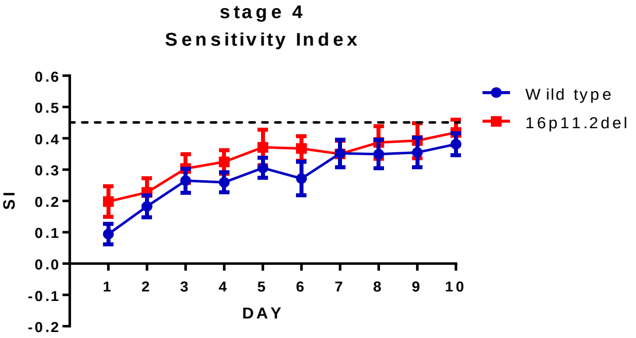

Mistakes - ANOVA: Main effect of genotype

day 6 (p = 0.052)
day 7 (p = 0.011)
day 8 (p = 0.011)
day 9 (p = 0.051)

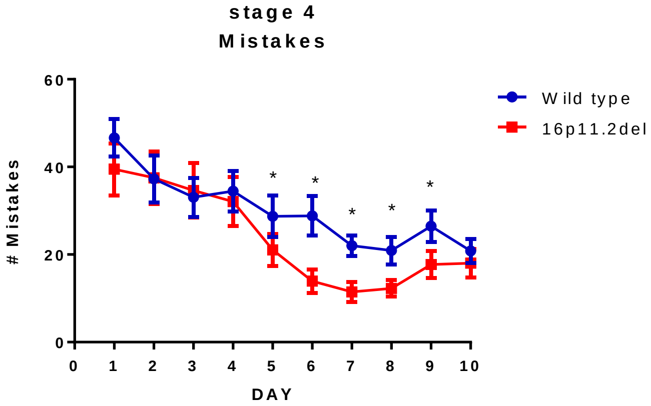

Supplementary Figure 2: Summary of the core limbic circuitry affected in male 16p11.2DEL mice. PFC/OFC: prefrontal /orbitofrontal cortex; Aud, Piri, Ins: auditory, piriform and Insular cortex; MS: medial septum; MB: mammillary bodies; Sub: subiculum; Amyg: basolateral amygdala; Acc: nucleus accumbens shell. Gray region names indicate unaltered metabolism. Gray arrows indicate unaltered connectivity.

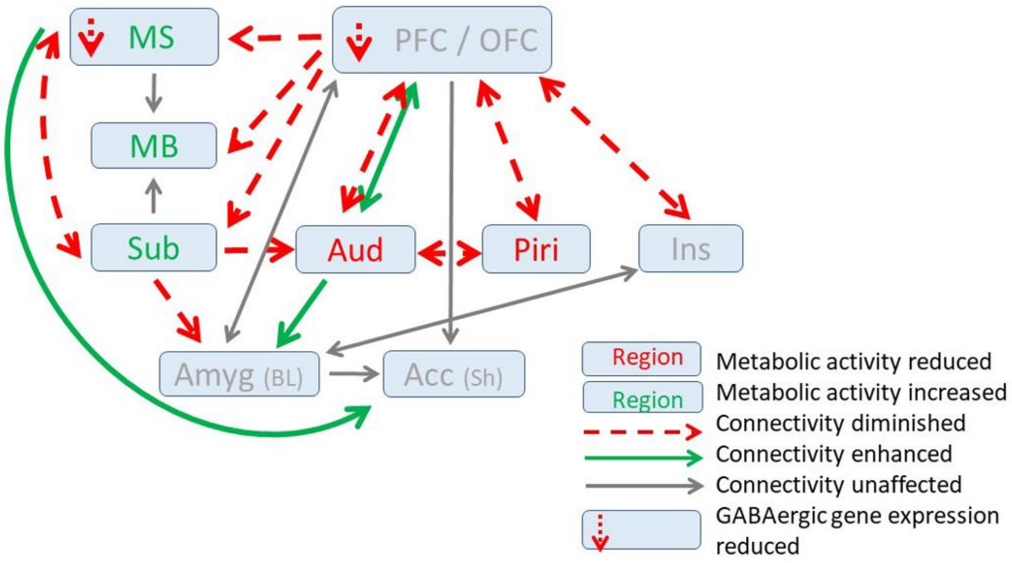

Supplement: Supplementary file 1 — Supplementary Information [file 42003_2023_4891_MOESM1_ESM.pdf]
